# The wider determinants of inequalities in health: a decomposition analysis

**DOI:** 10.1186/1475-9276-10-30

**Published:** 2011-07-26

**Authors:** Leonie Sundmacher, David Scheller-Kreinsen, Reinhard Busse

**Affiliations:** 1 Department of Health Care Management, Berlin University of Technology, H80, Strasse des 17. Juni 135, Berlin

## Abstract

**Background:**

The common starting point of many studies scrutinizing the factors underlying health inequalities is that material, cultural-behavioural, and psycho-social factors affect the distribution of health systematically through income, education, occupation, wealth or similar indicators of socioeconomic structure. However, little is known regarding if and to what extent these factors can assert systematic influence on the distribution of health of a population independent of the effects channelled through income, education, or wealth.

**Methods:**

Using representative data from the German Socioeconomic Panel, we apply Fields' regression based decomposition techniques to decompose variations in health into its sources. Controlling for income, education, occupation, and wealth, we assess the relative importance of the explanatory factors over and above their effect on the variation in health channelled through the commonly applied measures of socioeconomic status.

**Results:**

The analysis suggests that three main factors persistently contribute to variance in health: the capability score, cultural-behavioural variables and to a lower extent, the materialist approach. Of the three, the capability score illustrates the explanatory power of interaction and compound effects as it captures the individual's socioeconomic, social, and psychological resources in relation to his/her exposure to life challenges.

**Conclusion:**

Models that take a reductionist perspective and do not allow for the possibility that health inequalities are generated by factors over and above their effect on the variation in health channelled through one of the socioeconomic measures are underspecified and may fail to capture the determinants of health inequalities.

## Introduction

There is no shortage of empirical evidence illustrating the existence of health inequalities and association between socio-economic position and health inequalities is well established [1-3]. Reducing health inequalities, especially socioeconomic health inequalities, has therefore been on the agenda of policy-makers in a number of countries [4-6] and international organisations [7,8].

Nevertheless, the underlying mechanisms that determine health inequalities are not fully understood [9,10], which makes it hard for policy-makers to create well-targeted public policy strategies. On the conceptual level, various factors have been proposed to generate socioeconomic health inequalities including material factors, cultural-behavioural factors, and psycho-social factors [11,12]. Other important factors are ethnic- [13,14] and gender-based differences [15]. In health economics, the relative importance of these factors is commonly assessed by decomposition methods based on the concentration-index [16]. This process separates the contributions of individual factors to income-related health inequality, in which each contribution is the product of the sensitivity of health with respect to that factor and the degree of income-related inequality associated with that particular factor [16]. Various authors have contributed to this literature refining decomposition methods and their interpretation [17,18]. As an alternative to income-related health inequalities, education- [19], occupation- [1] and wealth-related health inequalities [20] have been assessed. Studies emerging from public health and epidemiology have used multiple regression analyses differentiated by education level or occupation [21-23] to assess the importance of different sets of factors [9]. A common starting point of both strands in the literature is that factors affecting health are rooted in or at least channelled through income, education, occupation, wealth or a similar indicator of socioeconomic structure. In the deepest sense, these approaches build on two schools of thought: the Marxian and the Weberian. In a very simplified framework the Marxian approach concentrates on the antagonistic class-relationship based on the distribution of means of production. In this framework, socioeconomic health inequalities emerge because of the different exposures to material factors, the most important being differences in work-related strains between the bourgeoisie and the working class. In the Weberian traditions classes are numerous and reflect the hierarchical structure in a number of dimensions such as prestige or status related to occupation, education, income, and other sources of power [24].

We use Fields' decomposition [25] techniques to decompose health inequalities, rather than socioeconomic inequalities in health, into their sources. This allows us to assess the relative importance of different sets of factors over and above their effect on the variation in health channelled through one of the measures of socioeconomic structure previously outlined. Our approach does not deny the especially worrisome nature of socioeconomic health inequalities, and nor do we suggest that income or other measures of socioeconomic structure are not import. Rather, we investigate if and to what extent different sets of factors can assert systematic influence on the distribution of health of a population independent of the effects channelled through income, education, or wealth. To our knowledge, no decomposition study has so far attempted to scrutinise the effect of different sets of explanatory factors over and above the influence channelled through income, education, and wealth.

Previous work on health inequalities in Germany were conducted from an epidemiological perspective within the framework of large, comparative European research projects [26-29]. In addition, income-related health inequalities were analysed in a comparative manner across countries applying approaches derived from health economists [30,31]. The particularities of health inequalities within Germany were discussed in various studies [32,33]. Overall, these studies suggest a moderate socio-economic gradient in health inequalities [34].

## Factors and Approaches

As outlined, different sets of explanatory factors have been proposed to account for health inequalities. We concentrate on three widely discussed sets of factors, namely, material, cultural-behavioural, and psycho-social factors, while also taking into account the life-course perspective. We also assess the capability approach as formulated by Hall and Taylor [35]. Due to limited space, we cannot fully develop the underlying theories and models, but various excellent overviews of the models and theories and their respective strengths and weaknesses exist and can be consulted [11,35].

The *materialist *approach explains health inequalities through differences in an individual's socio-economic position. The basic idea is that different social hierarchical positions in socio-economic stratification are linked to differential exposures to the material world, which can be either conducive or unconducive to health (e.g., noise, pollution, material working conditions). Various authors stressed that factors referring to the public infrastructure may determine the private resources available for health production and should also be considered as (neo-) material factors [36,37].

The *psycho-social approach *argues that individuals from lower socio-economic backgrounds experience more negative life events [38], less social support [39], less autonomy at work [40,41], less job security and therefore suffer from poorer health [42]. Various underlying mechanisms are assumed, but the core argument is that stress negatively affects health by reducing resilience and increasing vulnerability to illness [43]. Siegrist [44-46] puts forward a different variant of the psycho-social explanation, arguing that harmful stress is triggered by a perceived lack of reciprocity in the workplace, i.e., when rewards from employment or other central social roles are threatened or lost, persons become more vulnerable to addiction and other types of high risk behaviour due to biological processes in the brain [11].

The *cultural-behavioural *approach stresses that culture determines or frames behavioural choices, including decisions affecting health, i.e., engaging in higher risk lifestyles that may include drinking, smoking, or an unhealthy diet. Cultural-behavioural factors are often implicitly motivated by Bourdieu's concept of habitus [47]. Habitus is expressed in daily lifestyle decisions, partialities, body awareness, and consumption patterns. Differences in access to cultural, economic, and social capital are central to the class specific development of habitus patterns. In line with Bordieu's notion of habitus is the well-documented relationship between high educational attainment and health promoting behaviours.

The *life-course perspective *adds a temporal dimension and explains health inequalities as the result of differences in increasing and decreasing bundles of factors, which influence health at different times in an individual's life [48]. Thus, health is no longer solely the result of current conditions and individual lifestyle choices but is also determined by past living conditions and events [49].

Recently, Hall and Taylor [35] put forward the *capability approach*. Hall and colleagues follow the general analytical foundations formulated by Amartya Sen and others, arguing that the study of wellbeing, i.e., in our case 'health', should consider factors beyond material or narrow socioeconomic factors by focusing more broadly on the capabilities of people to realize functions (such as 'good health') they value [50,51]. This approach broadens the perspective on social relations as these can affect health independent of their relationship with people's income, employment, or wealth [35]. Hall et al. define an explicit micro-level explanatory mechanism and argue that an individual's health status is a function of individual capabilities and life challenges over time. Capabilities and challenges are, in turn, determined by socio-economic position, social connectedness, emotional disposition, collective imaginaries (understood as cultural and societal norms), self-determination, and stress. Hall and Taylor hypothesize that the observed socio-economic gradient in health inequalities is generated by differences in individual balances of capabilities and challenges.

## Data and Variables

The data for the analysis is taken from the 2006 German Socioeconomic Panel (GSOEP). The GSOEP is an annual panel with approximately 20,000 individuals aged 16 from over 11,000 households throughout Germany. Each person in the sample is interviewed individually. The GSOEP collects data on a broad range of issues, such as population and demography, education, training and qualification, earnings and income, health, basic orientation and satisfaction-specific aspects of life.

Our dependent variable physical health is measured through the reliable and validated Short Form-12 Health Survey (SF-12v2), which uses 12 questions to measure functional health and well-being from the individual's point of view and allows scores for physical and mental health to be generated.^1 ^Four subscales are used to generate a physical health score, and four are used to generate a mental health score respectively. The physical score mainly refers to evaluations of one's ability to perform physical activity. We use the physical health scores derived from the 2006 GSOEP as the dependent variable. As the discourses on mental health are very different [52], extending our analysis to mental health would require us to scrutinize an even greater variety of approaches. Therefore, we limit our analysis to physical health.

### Control Variables

To assess the relative importance of different sets of factors over and above their effect on the variation in health channelled through one of the socioeconomic measures commonly applied in decomposition analyses, we control for income, wealth, education and occupation. Wealth is measured using two binary variables: whether the person is a property owner and whether the person holds financial assets. To measure the impact of the level of wealth, we include two continuous variables: one for the monetary value of the property and one increasing in the value of financial assets. Occupation of the individual is operationalised using the Cambridge-Scale of Occupations. Education is captured using five binary variables that take a value of one when the individual has general elementary education, a mid-vocational, vocational or higher vocational qualification or higher education respectively. The reference cases are workers without any school qualification. To control for personal circumstances and working arrangement of each individual, the analysis also includes information on marital status, the number of children living in the household, whether the respondent has immigrant status, and whether the individual works part-time.

### Explanatory Variables

#### Material Factors

Working conditions are captured through a binary variable, which takes a value of one if the individual works in poor conditions. Moreover, we use an ordinal scale, five category variable on self-assessed pollution and noise, ranging from no impact to very strong impact and a binary variable on whether there is strong social coherence in the neighbourhood. We approximate infrastructural conditions by using two binary variables, which take a value of one if the individual needs more than twenty minutes to arrive at the doctor's practice or the nearest public transportation site and also include a self-assessed kilometre distance to the nearest big city. Differences caused by different exposures to the health care system are measured via the individual's mandatory, voluntary, or private health insurance arrangement.

#### Psychosocial-approach

To capture the psycho-social approach focusing on social support and the nature of living conditions, we include two binary variables, which take a value of one if the person lacks social or personal support (e.g., no one to confide in, no one to support his/her career). Furthermore, we use an ordinal variable increasing in perceived job security to approximate secure living conditions, an ordinal variable that captures the degree of job autonomy, and an ordinal variable for the level of the perceived time pressure at work. Siegrist's [44,45] reciprocity notion is conceptualized using interaction terms between the lifestyle variables of smoking and alcohol and a binary variable, which takes a value of one if the individual does not believe that his/her efforts at the workplace are adequately rewarded in terms of pay or direct appreciation.

#### Cultural-behavioural factor

We include four arguably reliable lifestyle variables from the 2006 GSOEP: how often the individual exercises, whether the individual smokes, whether the individual regularly drinks hard liquor, and how the individual's weight compares to the norm. The inclusion of the weight status is a proxy for the quantity and quality of food intake. To incorporate Bordieu's notion of culturally framed behaviours, we include further interactions between smoking and alcohol consumption and a binary variable on low educational attainment (lower or mid-vocation training) in the model.

#### Capability approach

The capability approach has not been operationalised for decomposition analysis of health inequalities yet. Our understanding is that an individual is equipped with resources that can be applied to life challenges. The discrepancy between the magnitude of resources enabling the capability to deal with negative life events and the sum of challenges decides the individual's ability to sustain good health. To capture the distance between resources and challenges, we calculate individual scores for resources and challenges and then subtract the latter from the former to generate one variable representing the capability approach. This approach departs from the original formulation by Sen and others by adding up several dimensions/variables of capabilities. It is, however, necessary to generate a measure that approximates what Hall and Taylor [35] consider the overall 'balance' between challenges and capabilities and its subsequent distribution across the population. Hence, our measure captures the combined effect of (different) capabilities and challenges, approximating the 'wear and tear' a person suffers in daily life [35].

Our resource score is computed by adding up the variables' social status (transformed in a five-category ordinal scale using quintiles), supportive confidantes (two binary variables), trust in democracy (a binary variable on whether the person belongs to the top 50 per cent of trustful citizens was calculated based on individuals' assessment on a ten-point scale ranging from zero (no trust) to ten (high trust)), and an ordinal variable capturing how well the person deals with stress on a seven-category scale.

The challenge score is constructed by adding up binary variables for poor working conditions, lack of advancement chances, lack of job security, and an ordinal variable increasing in lack of autonomy at work on a five-point scale.

### Modelling

We only include individuals 16 years of age and older who are employed either part- or full-time to a) test various theoretical explanations that explicitly draw on mechanisms based in working life and b) reduce potential endogeneity caused when people leave the working population due to health problems [31].^2 ^Excluding age and labour market status reduces the sample to 11,388. Of these individuals, 11,067 have a valid physical health score. After excluding the observations with missing values in the explanatory variables, 3,500 individuals in our final sample are female and 3,980 are male.

Previous research suggests that the determinants of health are different for males and females across age groups [53]. We therefore stratify the analysis by gender. Furthermore, the life-course approach suggests that different explanatory factors have different impacts at different points over the life-course. We adopt the life-course notion and stratify our cross-sectional data into four age groups: young (16-35), middle-aged one (36-45), middle-age two (46-55) and senior (56-65).^3 ^Nevertheless, we are aware that only by analysing different waves of panel-data could we truly investigate the differential or cumulative impact of factors over a person's life-course. As a result, our stratification strategy captures both age-specific and generation-specific effects. Each of the eight models is estimated using ordinary least squares with robust standard errors. The explained variance of the estimated models is then decomposed in factors.

## Methods

Research in the decomposition of factors is rooted in and driven by research applied to income inequality. In 1982, Shorrocks [54] developed a method that decomposes inequality in income by sources of factor components. Later, Murdoch and Sicular [55] and Fields [25] extended Shorrocks' [54] approach to a regression-based decomposition of inequality. They expressed household income as a linear function of explanatory variables and used the regression coefficients to calculate the decomposed variance for all variables in the model. The regression-based decomposition had the advantages that (1) it yielded an exact allocation of contributions to the identified factors, (2) it provided measures of uncertainty around the decomposed values that are part of standard regression analysis, and (3) it allowed for the analysis of multiple factors.

Given that our aim is to scrutinise the direct impact of various sets of explanatory variables on health, we choose to depart from the standard concentration index approach [56,2] and follow Fields' [25] decomposition method. Following Fields' method, health is first regressed on a range of explanatory variables using a standard least squares regression model of the form:(1)

where *Y_i _*is the health of individual i, *X_k _*is a vector of variables *X_1_, X_2_,...X_K _*thought to determine health (there are k = 1, 2, ... K variables included in *X_k_*), and *β_k _*is a vector of coefficients *β_1_, β_2_,... β_K _*pertaining to each variable k. *ε *is an error term with a mean value of zero and a variance of unity, *β_0 _*is the intercept term. The estimated coefficients are denoted by  and the residual term is given by 

To deduct the decomposition according to Fields [25], we then first take the variance of the left and right hand sides of equation (1), which is written as:(2)

Dividing (2) by the variance of Y then yields:(3)

The equation partitions the full variance of Y into the share that is explained by the covariance between each of the X factors and the Y values. Fields [25] calls the proportions denoted by *s(X_k_) *"relative factor inequality weights" or s-weights. It can be described as the share of the variance in health explained by the determinant k, holding all other determinants constant. Dividing the individuals' s-weights for each k by the model *R^2 ^*(the proportion of variance explained by all determinants *X_k _*taken together) gives the share of each factor in the explained variation of the linear regression, the so called p-weights.^4 ^Each p-weight, assigned to a variable k, gives the share of overall R-squared explained by this variable k. The sum of the p-weights is R-squared. The p-weights can therefore be interpreted as "little R-squared" for each of the variables k.

Formally, this is given by:(4)

Furthermore, Fields [25] shows that under six decomposition conditions (Additional File 1), the s-weights and p-weights are the same for any measure of dispersion that is continuous, symmetric, and takes value zero when all Y are identical (namely the Gini coefficient, the Theil index, and the Atkinson index).

Three points are important when interpreting the decomposition results. First, Fields' [25] approach decomposes the predicted value of Y rather than the actual value of Y. Thus, using this approach, we quantify the relative importance of determinants of explained inequality in Y. Second, Fields [25] also pointed out that the weights can take negative values.^5 ^Third, this method relies on the linearity of the model.

## Results

### Descriptive statistics

The level of inequality in physical health is descriptively quantified using two commonly used measures: the Gini coefficient and the Theil index. The Gini and Theil entropy measures illustrate that inequality in health increases with age. This is true for both men and women. However, the level of inequality is generally higher among females (Table 1).

**Table 1:** Inequality in physical health by age and sex

	Gini	Theil entropy	Gini	Theil entropy
	*Male *		*Female *	
16 to 34 years	0.057	0.006	0.126	0.069
35 to 44 years	0.074	0.010	0.143	0.078
45 to 54 years	0.087	0.013	0.169	0.093
Over 55 years	0.097	0.016	0.195	0.111

**Figure 1:**
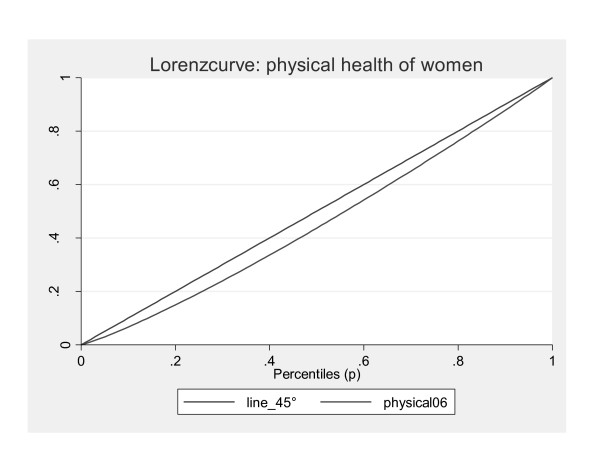
**Lorenz curves for women**.

**Figure 2:**
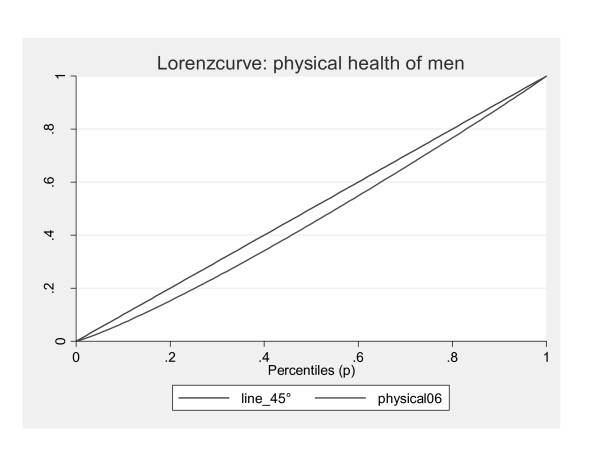
**Lorenz curves for men**.

Furthermore, the Theil index, which is more sensitive to inequality at the top of the distribution, has lower values indicating lower inequality among the healthier individuals. The Lorenz curve illustrates these findings graphically (Figures 1 and 2).

Summary statistics on all binary and continuous variables for women and men can be found in Table 2 and 3 respectively.

**Table 2:** Summary statistics for women

	16 to 35 years	36 to 44 years	45-55 years	55-65 years
Age	26.53 (4.87)	39.84 (2.81)	49.28 (2.82)	59.18 (3.91)
*Household income*				
1^st ^income quintile	11,861 (7449.54)	11,861 (7449.54)	13,657 (7742.27)	11,556 (7,421.08)
2^nd ^income quintile	32,210.4 (4285.76)	32,483.88 (3874.495)	32,640.79 (3934.83)	32057.35 (-4151.93)
3^rd ^income quintile	45,831.77 (4316.05)	46,844.98 (4536.895)	46,957.6 (4329.25)	47,010 (4262.40)
4^th ^income quintile	63,495.77 (6,492.74)	64,008.99 (6306.588)	64,698.61 (6345.57)	65,380.06 (6351.58)
5^th ^income quintile	107,327 (37,401.11)	112,829.1 (73,026.94)	115,820.6 (53,110.9)	119,078.7 (78,936.56)
*Education*				
No qualification	1%	0.78%	1.27%	1.19%
General elementary	15.27%	9.09%	8.92%	12.17%
Mid-vocational	46.89%	49.38%	47.23%	48.57%
Vocational	13.51%	9.41%	5.16%	2.39%
Higher vocational	6.60%	8.76%	7.52%	5.97%
Higher degree	16.73%	22.58%	29.89%	29.71%
Financial asset holder	45.59%	51.01%	54.63%	59.52%
Value of financial assets	3753 (10,313.59)	8192,447 (25,138.83)	15,925.04 (46,945.23)	20,987.77 (49,099.35)
Children living in Household (yes/no)	35.29%	68.44%	26.15%	2.20%
*Marital status*				
Married, separated	1.13%	3.24%	3.28%	1.96%
Single	67.87%	16.05%	6.88%	4.18%
Divorced	2.13%	12.94%	13.69%	12.27%
Married living together	25.87%	67.77%	76.16	81.59%

***Materialist approach***				
Poor working conditions	20.27%	26.04%	31.93%	22.87%
Noise level	1.84 (0.91)	1.86 (0.93)	1.88 (0.92)	1.81 (0.88)
Pollution level	1.72 (0.83)	1.73 (0.83)	1.73 (0.83)	1.66 (0.75)
Voluntary health insurance	7.39%	8.86%	8.74%	5.38%
Mandatory health insurance	66.69%	65.40%	65.75%	58.58%
More than 20 minute walk to nearest public transport	8.72%	11.23%	11.63%	12.37%
More than 20 minute walk to nearest GP practice	11.56%	12.55%	11.18%	15.79%

***Psychosocial approach ***				
Nobody to confide in	1.91%	3.14%	2.83%	5.13%
Nobody supports career	21.26%	39.58%	46.51%	55.43%
High autonomy at work	2.59 (0.88)	2.69 (0.96)	2.74 (1.04)	2.20 (1.07)
High time pressure at work	2.33 (0.87)	2.38 (0.85)	2.33 (0.89)	2.20 (0.94)
No job security	17.08%	15.65%	19.48%	12.95%

***Behavioural approach***				
Regular exercise 04	26.12%	30.94%	31.78%	28.92%
Occasional exercise 04	32.36%	32.22%	31.66%	32.44%
Smoking	34.18%	33.03%	29.48%	20.54%
Obese	7.19%	10.21%	15.47%	15.69%
Underweight	6.98%	2.46%	1.83%	0.59%
Regular consumption of hard liqueur	0.28%	0.19%	0.25%	0.45%
Smoking and low level of education	25.73%	24.14%	20.84%	12.77%
Smoking and lack of Recognition at the work place	22.27%	21.42%	18.48%	9.92%

**Table 3:** Summary statistics for men

	16 to 35 years	36 to 44 years	45-55 years	55 - 65 years
***Control variables***				
Age	26.70 (5.127)	39.73 (2.79)	49.27 (2.84)	59.90 (4.44)
*Household income*				
1^st ^income quintile	13,056.61 (7,710.17)	16,047 (7351.6)	15,945.88 (7507.82)	12,576.88 (7999.547)
2^nd ^income quintile	32,032.68 (4,001.038)	32,803 (4052.85)	32,787.09 (3904.39)	32,391.53 (3963.99)
3^rd ^income quintile	46,422.04 (4,495.192)	46,243 (4458.38)	46,829.55 (4577.21)	47,547.76 (4287.275)
4^th ^income quintile	63,406.36 (6,379.591)	64,153 (6166.71)	64,266.61 (6289.56)	65,178.31 (6470.122)
5^th ^income quintile	102,185 (48,842.48)	107,200 (33,486.01)	114,246.3 (48,723.55)	125,808.9 (92,624.47)
*Education*				
No qualification	2.02%	0.51%	0.72%	0.77%
General elementary	18.70%	7.27%	8.25%	8.16%
Mid-vocational	52.66%	45.25%	45.09%	37.89%
Vocational	8.00%	7.21%	3.93%	2.15%
Higher vocational	4.26%	13.44	9.95%	10.05%
Higher degree	14.36%	26.32%	32.07%	40.98%
Financial asset holder (yes/no)	42.49%	54.82%	57%	64.78%
Value of financial assets	5138.67 (18120)	13,728.9 (82,643.37)	19,518.3 (84,651)	43,626.8 (279,233.6)
Children living in Household	34.38%	64.04%	39.56%	8.47%
*Marital status*				
Married, separated	0.21%	2.21%	2.90%	2.96%
Single	74.19%	20.08%	7.47%	4.06%
Divorced	1.27%	7.13%	10.63%	8.81%
Married, living together	24.26%	70.36%	78.48%	81.88%

***Materialist approach***				
Poor working conditions	19.20%	28.45%	34.60%	25.06%
Noise level	1.868 (0.5453)	1.847 (0.9137)	1.856 (0.8959)	1.8369 (0.907)
Pollution level	1.732 (0.8215)	1.713 (0.8184)	1.740 (0.804)	1.6511 (0.768)
Voluntary health insurance	8.30%	16.58%	18.86%	19.05%
Mandatory health insurance	67.26%	59.14%	54.39%	42.68%
More than 20 minute walk to nearest public transport	8.85%	12.78%	10.95%	12.45%
More than 20 minute walk to nearest GP practice	12.79%	11.38%	11.51%	13.69%

***Psychosocial approach ***				
Nobody to confide in	5.58%	4.36%	4.57%	5.42%
Nobody supports career	23.42%	37.67%	46.52%	47.67%
High autonomy at work	2.4789 (1.039)	2.9931 (1.1017)	3.030 (1.1690)	3.288 (1.1753)
High time pressure at work	2.401 (0.8515)	2.546 (0.8482)	2.510 (0.8683)	2.2769 (0.8964)
No job security	17.63%	19.40%	21.07%	14.99%

***Behavioural approach***				
Regular exercise 04	29.07%	25.17%	26.05%	25.59%
Occasional exercise 04	29.41%	34.67%	33.46%	31.19%
Smoking	40.42%	36.79%	35.59%	24.41%
Obese	8.89%	15.52%	20.84%	21.25%
Underweight	1.44%	0.34%	0.19%	0.25%
Regular consumption of hard liqueur	10.78%	0.79%	1.10%	1.69%
Smoking and low level of education	33.56%	23.30%	23.05%	12.81%
Smoking and lack of Recognition at the work place	26.38%	36.79%	23.02%	11.44%

About 17 percent of females hold a higher education degree in the youngest age group (16 to 35 years). The rate increases to 30 percent in women aged 55 to 65 years. The rate is lower in men aged 16 to 35 years (ca. 14 percent) but considerably higher in the oldest age group (ca. 41 percent), illustrating a change in the gender-related difference in education between the generations. About 20 percent of women and men in the youngest age group work in poor conditions, compared to more than 30 percent of both sexes between 45 and 55 years of age. The feeling that nobody supports an individual's own career is quite similar in men and women (about 50 percent of the oldest age group), while the dissatisfaction is slightly higher among females. Obesity steadily increases with age in both sexes and peaks at in the oldest age group (15 percent of women and more than 20 percent of men). Smoking and a low level of education correlates for more than 20 percent of females and for more than 30 percent of males in the age group 16 to 35 years. This correlation decreases in the subsequent age groups.

### Decomposition results

In Table 4, we report the percentage contribution of each variable to the total sum of squares for women (i.e., the sum of the *s*-weights per factor of each of the competing approaches) by applying Fields' [25] regression techniques. The smoking status in the age group of 16 to 35 years of age explains, for example, approximately two percent of the total variance in health. Weight problems explain almost three percent of the total variation in health in individuals aged 56 to 65 years.

**Table 4:** Decomposition results by factors for women

	16 to 35 years	36 to 44 years	45 to 55 years	56 to 65 years
***Control variables***				
Household income	-0.0007	-0.0049	-0.0013	0.0171
Education	0.0194	0.0016	0.0042	0.0143
Occupation	0.0007	0.0019	0.0012	-0.0033
House ownership and value of property	0.0005	0.0004	0.0002	0.0017
Financial assets and value of financial assets	0.0010	0.0057	0.0004	0.0290
Children	0.0002	0.0022	0.0048	0.0008
Marital status	0.0174	0.0015	-0.0001	0.0066
Immigrant status	0.0015	0.0005	0.0065	0.0035
Part-time work	0.0008	0.0006	0.0000	0.0003

***Materialist approach***				
Poor working conditions	-0.0007	0.0020	0.0000	0.0227
Noise	0.0055	-0.0006	0.0054	0.0001
Pollution	0.0007	0.0007	0.0002	0.0002
Insurance status	0.0005	0.0009	0.0024	0.0016
Distance to nearest doctors practice, public transport and big city	0.0012	0.0004	-0.0005	-0.0002

***Psychosocial approach ***				
Nobody to confide in	0.0007	0.0048	0.0015	0.0002
Nobody supports career	0.0006	-0.0003	0.0042	0.0006
Degree of autonomy at work	0.0042	0.0190	0.0061	0.0025
Time pressure at work	0.0006	0.0002	-0.0004	0.0024
Level of job security	0.0008	-0.0021	-0.0007	-0.0041
Smoking and a lack of recognition at the workplace	-0.0024	0.0016	0.0005	0.0032
Alcohol and a lack of recognition at the workplace	0.0057	0.0001	0.0013	0.0022

***Cultural behavioural approach ***				
Exercise	0.0060	0.0069	0.0083	0.0140
Hard-liqueur consumption	0.0001	0.0005	0.0002	0.0038
Smoking status	0.0197	-0.0010	0.0030	0.0061
Weight problems	0.0551	0.0181	0.0279	0.0273
Smoking interacted with low education	-0.0092	0.0025	0.0003	0.0052
Alcohol interacted with low education	0.0054	0.0004	0.0000	0.0003

***Capability approach***	0.0046	0.0374	0.0334	0.0618
Observations	701	1112	1150	537
R-squared	0.14	0.10	0.11	0.22

**Figure 3:**
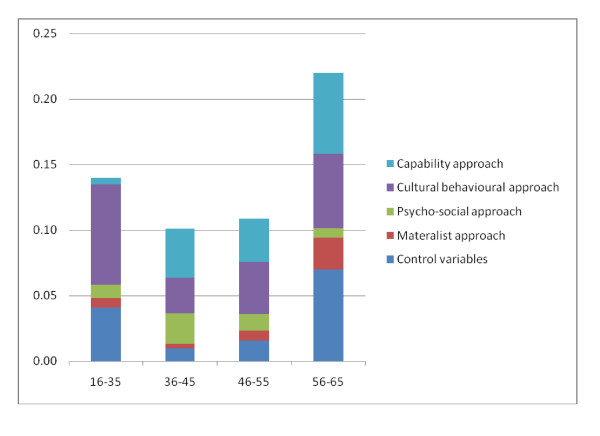
**Relative importance of different approaches to explain health inequalities - women**.

Figure 3 illustrates the relative importance of the different approaches towards health inequalities in the four age groups. The height of the bar indicates the overall explained sum of squares. It ranges between approximately 10 (in the age group 36 to 45 years) and 22 percent (in the oldest age group).

Cultural-behavioural variables are most influential in the youngest generation (16 to 35 years). In the subsequent two age groups, their relevance in terms of total R-squared is lower but increases again in the fourth age group. Other variables, such as material variables and the capability score become relevant in later age groups. The variables summarised under the material perspective play a prominent role in the last age group, but their impact is low between the ages of 16 and 55. The capability score (i.e., the distance between challenges and resources) is a strong and increasing explanatory factor between 35 and 65 years but is far less important to the first age group. The contribution of psycho-social variables is substantial in the second age group. For all other age groups, the contributions are modestly relevant. The importance of the control variables is high in the first and last age group, but it contributes to explained variance on a low level between 36 and 55 years of age.

In Table 5, the explained variance of health steadily increases in the first three age groups (from about 10 percent to 18 percent) but slightly decreases in the last age group (to about 16 percent).

**Table 5:** Decomposition results by factors for men

	16 to 35 years	36 to 44 years	45 to 55 years	56 to 65 years
***Control variables***				
Household income	0.0021	-0.0014	0.0114	0.0087
Education	0.0025	0.0261	0.0138	0.0084
Occupation	0.0035	0.0069	0.0156	-0.0008
House ownership and value of property	0.0053	0.0015	-0.0018	0.0000
Financial assets and value of financial assets	0.0014	0.0109	0.0073	-0.0008
Children	-0.0006	0.0016	0.0147	0.0018
Marital status	0.0019	0.0079	0.0103	0.0081
Immigrant status	0.0036	0.0008	-0.0003	0.0007
Part-time work	0.0019	0.0000	0.0017	0.0007

***Materialist approach***				
Poor working conditions	-0.0011	0.0088	0.0069	0.0070
Noise	0.0074	-0.0002	0.0062	0.0025
Pollution	0.0001	0.0016	0.0007	0.0029
Insurance status	0.0007	0.0055	0.0032	0.0027
Distance to nearest doctors practice, public transport and big city	0.0002	-0.0003	-0.0010	0.0034

***Psychosocial approach***				
Nobody to confide in	0.0002	-0.0001	0.0000	0.0020
Nobody supports career	0.0048	0.0006	0.0005	0.0085
Degree of autonomy at work	0.0015	0.0013	0.0108	-0.0026
Time pressure at work	0.0041	-0.0008	-0.0009	-0.0012
Level of job security	0.0007	0.0032	0.0036	-0.0028
Smoking and a lack of recognition at the workplace	0.0074	0.0040	-0.0061	-0.0005
Alcohol and a lack of recognition at the workplace	0.0006	0.0018	0.0033	0.0009

***Cultural behavioural approach***				
Exercise	-0.0004	0.0061	0.0102	0.0160
Hard-liqueur consumption	0.0134	0.0022	0.0003	0.0015
Smoking status	-0.0019	-0.0018	0.0130	0.0000
Weight problems	0.0097	0.0080	0.0145	0.0306
Smoking interacted with low education	0.0066	0.0033	-0.0006	0.0006
Alcohol interacted with low education	-0.0006	0.0000	-0.0015	0.0072

***Capability approach***	0.0207	0.0219	0.0396	0.0511
Observations	715	1291	1180	794
R-squared	0.10	0.12	0.18	0.16

For men, behavioural variables contribute to variance in health to a substantial degree. The effects are largest in the last two age groups. The capability approach also exerts a considerable effect on total R-squared. It relatively accounts for the explained variance in health more or less equally in the first two age groups but has a high contribution for the last two age groups. Total contribution to R-squared is highest in the fourth age group. The psycho-social approach is most influential in the first age group, and the impact varies between low and medium contribution for the other age groups.

**Figure 4:**
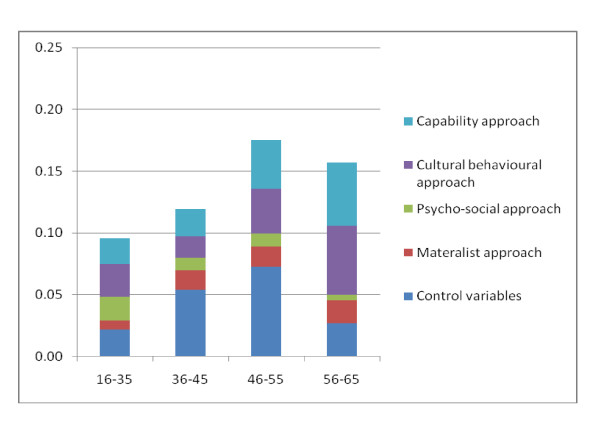
**Relative importance of different approaches to explain health inequalities - men**.

The importance of the control variables steadily increases between the ages of 45 and 55 and is the most influential factor in the third age group. In fact, control variables contribute more than seven percent to the explanation of the total sum of squares. Interestingly, the control variables play a less important role for the last age group. Figure 4 illustrates the contributing factors by approach.

Each of the decompositions is based on an ordinary regression model, which provides information on the direction and magnitude of the coefficients. In the regression models, behavioural variables (obesity in females and hard liqueur consumption in men) have a comparable high statistical siginificance at young age. The same holds for obesity in the older age groups as it has a direct impact on health.^6 ^The capabaility variable is statistically significant throughout the models. All coefficients of the underlying regression models for women and men used in the decomposition can be found in Additional File 2.

## Discussion

Scrutinising the results of the analysis allows us to deduce informed hypotheses about mechanisms underlying health inequalities. The analysis suggests that three main factors persistently contribute to variance in health over and above their effect on the variation in health channelled through one of the socioeconomic measures: the capability score, cultural-behavioural variables, and to a lower extent the materialist approach.

Of the three, the capability score illustrates the importance of interaction and compound effects as it captures the individual's socioeconomic, social, and psychological resources in relation to his/her exposure to life challenges. On one hand, the high explanatory power of the variable suggests that independent challenges for health (e.g., low household income or education) can be tackled if the individual has access to a high level of resources (e.g., high social capital). On the other hand, it illustrates the difficulties of maintaining good health if the individual faces many challenges while resources are low. Moreover, our analysis shows that for men and women, the difference between resources and challenges is particularly important during the last stage of work life (46 to 65 years). This may suggest that individuals become increasingly vulnerable when they face a high disparity of challenges and resources over a long time or in older age. Individuals may for example feel more strained as the challenges posed by job and family life persist, but the efficiency in the production of their own health decreases, and therefore, the ability to maintain good health decreases.

The results for the materialist variables complement the findings for the capability score. Our analysis for women and men proposes that materialist variables are important for health inequalities during the last stage of work life. However, considering the relative importance of the capability score compared to materialist variables, this perspective suggests that for old age, inequalities are even better captured if one considers vulnerability in terms of the interacted/compound effects of the discrepancy between resources and challenges.

With regard to the cultural-behavioural variables, our analysis suggests that these increasingly contribute to R-squared for individuals from older age groups (36 to 65 years). Therefore, being overweight is a key contributor to health inequalities for both sexes during later stages of work life. Moreover, our analysis shows that cultural-behavioural variables are important in the youngest generation, especially for women. Again we see that weight problems contribute to health inequalities in women while the consumption of hard liqueur explains a high share of variation in health in men.

The control variables add explanatory power across all models and especially in the first and last age group for women and in the middle age groups for men. Therefore, they should be considered in fully specified analyses.

There are a number of limitations to our analysis. First, we do not claim that the selection of variables exhaustively reflects the notions of the presented theories. Building on previous studies, we try to operationalise the approaches as closely as possible, but some choices in operationalisation will remain ultimately normative. For example, we did not include the interaction between smoking and low levels of household income in the decomposition analysis because we considered the cultural-behavioural effect to be better captured by the interaction between smoking and low levels of education. Although this view was informed by our literature review, the decision remains subjective.

Second, the approaches are operationalised with different numbers of variables. This might marginally affect the contribution to variance in the analysis. However, we are confident that this shortcoming is moderate. For example the capability approach, which is operationalised with one variable, is a major contributor in the analysis. Overall, our aim is to give an impression of the relative importance of the variables. We do not claim that the relative percentages should be taken at face value because they might be slightly distorted by collinearity. Last, in an econometric sense we do not apply methods that allow conclusions about causal mechanisms. Therefore we have to be cautious not to overstate our results.

## Conclusions

Models that take a reductionist perspective and do not allow for the possibility that health inequalities are generated by factors over and above their effect on the variation in health channelled through one of the socioeconomic measures are underspecified and may fail to capture the determinants of health inequalities. This was particularly evident when we modelled the distance between resources and challenges in life, which in our analysis is a very important factor contributing to inequality in health.

## Competing interests

The authors declare that they have no competing interests.

## Authors' contributions

LS participated in the design and concept of the study, carried out the statistical analysis, and drafted the manuscript. DSK participated in the design of the study, reviewed the literature and drafted the manuscript. RB participated in the revision of the manuscript and approved the final manuscript. All authors read and approved the final manuscript.

## Endnotes

^1 ^The GSOEP version of the SF-12v2 deviates from the original SF-12v2 to a limited degree with regard to formulation, order of questions, and general layout. See Anderson et al. for more specific information (2007: 172).

^2 ^We chose an age of 16 as the lower cut-off point for inclusion in the sample because 16 is the minimum age for legal work in Germany. About 20% of youths between the age of 16 and 18 are in the labour force.

^3 ^We believe that the working population over age 65 represents a selected sample of likely very healthy individuals. To avoid any distortions in the analysis, we decided to use the official retirement age as the upper cut-off for the last age group.

^4 ^Note that  is the model *R^2^*.

^5 ^He explained this result as follows: the covariance between  and Y can be expressed as  and when we regress of Y on *X_k_*, we obtain the simple regression coefficient . It follows that the p-weights can be written as . This implies that a negative value arises whenever the two beta coefficients have opposite signs (i.e., whenever controlling for multiple factors within a regression framework would reverse the sign from the simple regression).

^6 ^The significance level of the single coefficient of dummy variables (household income, education, insurance etc.) in the regression models is low. However their combined effect (e.g., five dummy variables for education) is most of the time significant (using a F-test).

## Supplementary Material

Additional file 1 **A1. Decomposition conditions**. Lists six conditions (based on Shorrocks (1982, 1983) and Fields (2004)) in which the s-weights and p-weights are the same for any measure of dispersion that is continuous, symmetric, and takes the value zero when all Y are identical (the Gini coefficient, Theil index, and Atkinson index). Click here for file

Additional file 2 **A2. Regression results**. Shows the results from the four regression models (A2.1 - A2.4) underlying the decompositions. Click here for file
