# Supplementary material for: The wider determinants of inequalities in health: a decomposition analysis
Source: Int J Equity Health. 2011 Jul 26;10:30. doi: 10.1186/1475-9276-10-30 (PMC3171309; doi:10.1186/1475-9276-10-30)
Supplement: Additional file 1 — A1. Decomposition conditions. Lists six conditions (based on Shorrocks (1982, 1983) and Fields (2004)) in which the s-weights and p-weights are the same for any measure of dispersion that is continuous, symmetric, and takes the value zero when all Y are identical (the Gini coefficient, Theil index, and Atkinson index). [file 1475-9276-10-30-S1.DOC]

***A1. Decomposition conditions***

The following are six conditions (based on Shorrocks (1982, 1983) and Fields (2004)) in which the s-weights and p-weights are the same for any measure of dispersion that is continuous, symmetric, and takes the value zero when all Y are identical (the Gini coefficient, Theil index, and Atkinson index):

1. The inequality measure I(Y) is to be divided into K components, one for each regressors, denoted by $S_{k}=S_{k}(Y^{1},...,Y^{k};K)$
2. Each $S_{k}$ is continuous in $Y_{k}$.
3. The amount of inequality accounted for by any one factor $S_{k}$does not depend on how the other factors are grouped, i.e. $S_{k}(Y^{1},...,Y^{k};K)=S_{k}(Y^{k},Y)$
4. The contribution of $S_{k}$sum to the overall amount of inequality
5. If P is any n * n permutation matrix, then$S(Y^{k}P,YP)=S(Y^{k},Y)$. Furthermore, if all individuals i have the same value for the k’th factor, then the share of inequality accounted for by that factor is $S_{k}(\mu_{k}e,Y)=0$ for $\mu_{k}$.
6. Suppose the distribution of Y1 is only a permutation of Y2. If Y1 and Y2 are the only two components in the decomposition, then they should receive the same share in the decomposition.
