# Supplementary material for: The wider determinants of inequalities in health: a decomposition analysis
Source: Int J Equity Health. 2011 Jul 26;10:30. doi: 10.1186/1475-9276-10-30 (PMC3171309; doi:10.1186/1475-9276-10-30)
Supplement: Additional file 2 — A2. Regression results. Shows the results from the four regression models (A2.1 - A2.4) underlying the decompositions. [file 1475-9276-10-30-S2.DOC]

***A2. Regression results***

Table A2.1: Regression results for women aged 16 to 44 years

|  | **16 to 35 years** |  | **36 to 44 years** |  |
| --- | --- | --- | --- | --- |
| **Women** | **Coefficients** | **p-values** | **Coefficients** | **p-values** |
| Separated | -4.4614 | 0.0860 | -0.1280 | 0.5360 |
| Single | 1.7703 | 0.0120 | 0.8282 | 0.5210 |
| Divorced | 2.6949 | 0.2040 | 0.5538 | 0.5860 |
| Number of children | 0.7249 | 0.0720 | 0.7301 | 0.1160 |
| Immigrant status | 1.3261 | 0.1750 | -1.7587 | 0.7630 |
| Part-time work | -0.3184 | 0.6340 | -0.3415 | 0.4190 |
| General elementary | 0.7550 | 0.8570 | 3.7212 | 0.6440 |
| Mid-vocational | 2.9845 | 0.4700 | 3.9742 | 0.6460 |
| Vocational | 4.4700 | 0.2880 | 1.4438 | 0.9960 |
| Higher vocational | 3.2721 | 0.4390 | 2.6316 | 0.7500 |
| Higher degree | 4.3403 | 0.3050 | 3.4189 | 0.6880 |
| 1^st^ income quintile | 0.9317 | 0.4470 | 0.3030 | 0.0310 |
| 2^nd^ income quintile | 1.1459 | 0.3140 | 0.7625 | 0.0420 |
| 3^rd^ income quintile | 1.0032 | 0.3770 | 1.2561 | 0.3260 |
| 4^th^ income quintile | 0.7438 | 0.5000 | 0.4150 | 0.5090 |
| Poor working conditions | 0.5856 | 0.4460 | -0.0054 | 0.4890 |
| Prestige | 0.0025 | 0.8630 | 0.0027 | 0.7010 |
| House-ownership (0/1) | 0.8120 | 0.5330 | 0.5964 | 0.9000 |
| Value of property | 0.0000 | 0.4490 | 0.0000 | 0.3090 |
| Financial assets (0/1) | -0.2716 | 0.6470 | 0.1956 | 0.0570 |
| Value of financial assets | 0.0000 | 0.4640 | 0.0000 | 0.5430 |
| Regular exercise 04 | -0.3911 | 0.5840 | 1.0218 | 0.8680 |
| Occasional exer­cise 04 | -1.3810 | 0.0380 | -0.3505 | 0.0080 |
| Consumption of hard liqueur | -0.9426 | 0.8240 | 1.9292 | 0.5270 |
| Smoking Status | -3.4642 | 0.0050 | -0.7801 | 0.7850 |
| Obesity | -5.6819 | 0.0000 | -3.5155 | 0.0000 |
| Underweight | -2.6245 | 0.0260 | 2.9524 | 0.5450 |
| Smoking * lack of recognition | 0.7915 | 0.4370 | -0.1370 | 0.5940 |
| Alcohol * lack of recognition | 3.1823 | 0.1270 | 1.7790 | 0.8530 |
| Smoking * low edu­cational level | 2.5320 | 0.0330 | -0.1069 | 0.5190 |
| Alcohol * low educational level | 3.7173 | 0.1630 | 0.0910 | 0.7000 |
| Mandatory health insurance | 0.0137 | 0.9850 | 1.1067 | 0.1960 |
| Voluntary health insurance | 1.0886 | 0.3120 | 0.0963 | 0.1660 |
| More than 20 minute walk to nearest GP practice (0/1) | 0.3099 | 0.7020 | -0.1936 | 0.2010 |
| More than 20 minute walk to nearest public transport (0/1) | -0.5091 | 0.8220 | -2.5331 | 0.8640 |
| Distance to nearest big city | 0.1033 | 0.5840 | -0.0445 | 0.5360 |
| Noise level | -0.4941 | 0.2340 | -0.6236 | 0.5860 |
| Pollution level | -0.1109 | 0.8060 | -0.0292 | 0.5790 |
| Have nobody to confide in (0/1) | -1.1104 | 0.6380 | -1.8690 | 0.0250 |
| Nobody supports career | 0.7587 | 0.2390 | -0.7397 | 0.1160 |
| Autonomy at work | 0.2933 | 0.4860 | 0.3027 | 0.0080 |
| Time pressure at work | -0.2224 | 0.5450 | 0.1635 | 0.9200 |
| No job security | -0.3284 | 0.6860 | 0.2415 | 0.1600 |
| Capability score | 0.1556 | 0.3150 | 0.4937 | 0.0000 |
| Constant | 48.6769 | 0.0000 | 43.8647 | 0.0000 |
| Observation | 701 |  | 1112 |  |
| R-squared | 0.14 |  | 0.10 |  |

Tab. A1

Table A2.2: Regression results for women aged 45 to 65 years

|  | **45 to 55 years** |  | **56 to 65 years** |  |
| --- | --- | --- | --- | --- |
| **Women** | **Coefficients** | **p-values** | **Std. errors** | **p-values** |
| Separated | -0.1280 | 0.9310 | 3.6083 | 0.2110 |
| Single | 0.8282 | 0.4290 | -0.0020 | 0.9990 |
| Divorced | 0.5538 | 0.4810 | 2.0618 | 0.1220 |
| Number of children | 0.7301 | 0.0510 | 1.6654 | 0.4200 |
| Immigrant status | -1.7587 | 0.0580 | -1.0019 | 0.4120 |
| Part-time work | -0.3415 | 0.5110 | -0.2962 | 0.7030 |
| General elementary | 3.7212 | 0.1790 | 3.0087 | 0.4310 |
| Mid-vocational | 3.9742 | 0.1440 | 2.7034 | 0.4720 |
| Vocational | 1.4438 | 0.6180 | 8.4553 | 0.0480 |
| Higher vocational | 2.6316 | 0.3570 | 4.3033 | 0.2860 |
| Higher degree | 3.4189 | 0.2200 | 3.3849 | 0.3760 |
| 1^st^ income quintile | 0.3030 | 0.7850 | -1.7905 | 0.2620 |
| 2^nd^ income quintile | 0.7625 | 0.4240 | -0.7145 | 0.6410 |
| 3^rd^ income quintile | 1.2561 | 0.1410 | -0.2151 | 0.8820 |
| 4^th^ income quintile | 0.4150 | 0.5780 | -2.2521 | 0.0920 |
| Poor working conditions | -0.0054 | 0.9940 | -3.0085 | 0.0050 |
| Prestige | 0.0027 | 0.8260 | -0.0107 | 0.5320 |
| House-ownership (0/1) | 0.5964 | 0.3550 | -1.2604 | 0.1870 |
| Value of property | 0.0000 | 0.2130 | 0.0000 | 0.3390 |
| Financial assets (0/1) | 0.1956 | 0.7160 | 1.1981 | 0.1650 |
| Value of financial assets | 0.0000 | 0.6130 | 0.0000 | 0.0080 |
| Regular exercise 04 | 1.0218 | 0.1120 | 2.0000 | 0.0410 |
| Occasional exer­cise 04 | -0.3505 | 0.5810 | 0.8143 | 0.4010 |
| Consumption of hard liqueur | 1.9292 | 0.6420 | 9.0772 | 0.1420 |
| Smoking Status | -0.7801 | 0.4810 | 1.5175 | 0.3790 |
| Obesity | -3.5155 | 0.0000 | -3.4390 | 0.0010 |
| Underweight | 2.9524 | 0.0920 | -1.0393 | 0.8110 |
| Smoking * lack of recognition | -0.1370 | 0.8860 | -4.1763 | 0.0160 |
| Alcohol * lack of recognition | 1.7790 | 0.3060 | -2.2543 | 0.4120 |
| Smoking * low edu­cational level | -0.1069 | 0.9260 | 2.6551 | 0.1680 |
| Alcohol * low educational level | 0.0910 | 0.9640 | -0.5770 | 0.8560 |
| Mandatory health insurance | 1.1067 | 0.0760 | 1.5484 | 0.0890 |
| Voluntary health insurance | 0.0963 | 0.9210 | 2.0373 | 0.2420 |
| More than 20 minute walk to nearest GP practice (0/1) | -0.1936 | 0.8020 | -0.4493 | 0.6480 |
| More than 20 minute walk to nearest public transport (0/1) | -2.5331 | 0.1880 | -0.5145 | 0.8710 |
| Distance to nearest big city | -0.0445 | 0.7990 | -0.1908 | 0.4640 |
| Crime level | 0.1319 | 0.7810 | 0.2333 | 0.7490 |
| Noise level | -0.6236 | 0.0840 | -0.1297 | 0.8160 |
| Pollution level | -0.0292 | 0.9420 | 0.5232 | 0.4620 |
| Have nobody to confide in (0/1) | -1.8690 | 0.2160 | -0.4890 | 0.7870 |
| Nobody supports career | -0.7397 | 0.1450 | -0.2609 | 0.7290 |
| Autonomy at work | 0.3027 | 0.4160 | 0.1485 | 0.7840 |
| Time pressure at work | 0.1635 | 0.6210 | -0.1872 | 0.7250 |
| No job security | 0.2415 | 0.7470 | 3.0995 | 0.0140 |
| Capability score | 0.4937 | 0.0010 | 0.6029 | 0.0090 |
| Constant | 43.8647 | 0.0000 | 41.5285 | 0.0000 |
| Observation | 1150 |  | 537 |  |
| R-squared | 0.11 |  | 0.22 |  |

Tab. A2

Table A2.3: Regression results for men aged 16 to 44 years

|  | **16 to 35 years** |  | **36 to 44 years** |  |
| --- | --- | --- | --- | --- |
| **Men** | **Coefficients** | **p-values** | **Coefficients** | **p-values** |
| Separated | 5.1482 | 0.1330 | 1.5039 | 0.3330 |
| Single | -0.4973 | 0.4430 | 1.7552 | 0.0050 |
| Divorced | -1.3491 | 0.4870 | 0.9057 | 0.2850 |
| Number of children | 0.1130 | 0.7360 | 0.5720 | 0.0110 |
| Immigrant status | -0.9705 | 0.2010 | -0.2724 | 0.6730 |
| Part-time work | -0.7593 | 0.2730 | 0.0547 | 0.9450 |
| General elementary | 2.0642 | 0.2830 | 2.0794 | 0.4800 |
| Mid-vocational | 2.0067 | 0.2750 | 3.1516 | 0.2750 |
| Vocational | 2.3130 | 0.2560 | 2.7637 | 0.3590 |
| Higher vocational | 1.0400 | 0.6250 | 3.3350 | 0.2630 |
| Higher degree | 1.7735 | 0.3800 | 4.7690 | 0.1090 |
| 1^st^ income quintile | 1.6952 | 0.1030 | 0.3628 | 0.7240 |
| 2^nd^ income quintile | 0.2295 | 0.7990 | 0.4279 | 0.6020 |
| 3^rd^ income quintile | 0.0020 | 0.9980 | 0.7135 | 0.3210 |
| 4^th^ income quintile | 0.1981 | 0.8170 | 0.6261 | 0.3430 |
| Poor working conditions | 0.3032 | 0.6410 | -1.0327 | 0.0540 |
| Prestige | 0.0064 | 0.5930 | 0.0077 | 0.3790 |
| House-ownership (0/1) | -1.0571 | 0.2030 | -0.6553 | 0.1880 |
| Value of property | 0.0000 | 0.8730 | 0.0000 | 0.6120 |
| Financial assets (0/1) | 0.1843 | 0.7010 | 0.8917 | 0.0370 |
| Value of financial assets | 0.0000 | 0.6900 | 0.0000 | 0.3810 |
| Regular exercise 04 | -0.4147 | 0.4760 | 0.8950 | 0.0900 |
| Occasional exer­cise 04 | -0.2733 | 0.6210 | 0.2186 | 0.6480 |
| Consumption of hard liqueur | -6.2321 | 0.0020 | -4.3370 | 0.0820 |
| Smoking Status | 2.3040 | 0.0240 | 0.4256 | 0.5760 |
| Obesity | -1.7149 | 0.0300 | -1.3353 | 0.0120 |
| Underweight | 1.2089 | 0.5490 | -2.9352 | 0.3980 |
| Smoking * lack of recognition | -1.6646 | 0.0310 | -0.6945 | 0.3330 |
| Alcohol * lack of recognition | -0.2352 | 0.7870 | -0.6101 | 0.3890 |
| Smoking * low edu­cational level | -1.5184 | 0.1400 | -0.4926 | 0.5540 |
| Alcohol * low educational level | 0.2523 | 0.7770 | -0.0122 | 0.9880 |
| Mandatory health insurance | 0.2190 | 0.7220 | -0.3246 | 0.5620 |
| Voluntary health insurance | -1.2748 | 0.1460 | -0.2605 | 0.6730 |
| More than 20 minute walk to nearest GP practice (0/1) | 0.0360 | 0.9550 | 0.2533 | 0.6580 |
| More than 20 minute walk to nearest public transport (0/1) | 0.5087 | 0.7670 | 1.3827 | 0.4020 |
| Distance to nearest big city | 0.1364 | 0.3690 | -0.3058 | 0.0260 |
| Crime level | -0.1290 | 0.7660 | 0.0791 | 0.8330 |
| Noise level | 0.7097 | 0.0380 | 0.1019 | 0.7450 |
| Pollution level | -0.4402 | 0.2480 | -0.4075 | 0.2540 |
| Have nobody to confide in (0/1) | 0.4134 | 0.6960 | 0.4335 | 0.6620 |
| Nobody supports career | -0.7083 | 0.1530 | 1.0462 | 0.0110 |
| Autonomy at work | 0.0733 | 0.8170 | 0.0474 | 0.8600 |
| Time pressure at work | 0.6850 | 0.0310 | 0.2429 | 0.3930 |
| No job security | -0.1282 | 0.8560 | -0.5042 | 0.3900 |
| Capability score | 0.3640 | 0.0100 | 0.2602 | 0.0350 |
| Constant | 49.7600 | 0.0000 | 46.8228 | 0.0000 |
| Observation | 715 |  | 1291 |  |
| R-squared | 0.10 |  | 0.12 |  |

Tab. A3

Table A2.4: Regression results for men aged 45 to 65 years

|  | **45 to 55 years** |  | **56 to 65 years** |  |
| --- | --- | --- | --- | --- |
| **Men** | **Coefficients** | **p-values** | **Coefficients** | **p-values** |
| Separated | 2.4555 | 0.0930 | -0.9558 | 0.5660 |
| Single | 3.5977 | 0.0000 | 2.0373 | 0.1930 |
| Divorced | 1.1909 | 0.1200 | 2.9405 | 0.0090 |
| Number of children | 0.8704 | 0.0000 | 0.7246 | 0.3390 |
| Immigrant status | 0.6380 | 0.4060 | -0.4230 | 0.6870 |
| Part-time work | -0.7585 | 0.3790 | -0.6215 | 0.5300 |
| General elementary | 1.2043 | 0.6210 | -2.7761 | 0.5820 |
| Mid-vocational | -0.1397 | 0.9530 | -2.0509 | 0.6790 |
| Vocational | 1.6560 | 0.5250 | 2.7757 | 0.6020 |
| Higher vocational | -0.8051 | 0.7470 | -1.7669 | 0.7270 |
| Higher degree | 0.8016 | 0.7450 | -1.7217 | 0.7320 |
| 1^st^ income quintile | -1.8447 | 0.1280 | -0.7510 | 0.5740 |
| 2^nd^ income quintile | -0.8229 | 0.3820 | 0.0841 | 0.9420 |
| 3^rd^ income quintile | 0.0886 | 0.9120 | -1.4045 | 0.1820 |
| 4^th^ income quintile | -0.0480 | 0.9410 | 0.0352 | 0.9660 |
| Poor working conditions | -0.8009 | 0.1530 | -1.1455 | 0.1380 |
| Prestige | 0.0174 | 0.0850 | -0.0014 | 0.9050 |
| House-ownership (0/1) | 0.3369 | 0.5710 | 0.6621 | 0.3640 |
| Value of property | 0.0000 | 0.3810 | 0.0000 | 0.3120 |
| Financial assets (0/1) | 0.8552 | 0.0710 | -0.6534 | 0.3120 |
| Value of financial assets | 0.0000 | 0.9030 | 0.0000 | 0.6910 |
| Regular exercise 04 | 1.2080 | 0.0360 | 1.9134 | 0.0120 |
| Occasional exer­cise 04 | 0.4365 | 0.4090 | 0.9860 | 0.1530 |
| Consumption of hard liqueur | 2.0442 | 0.3640 | 2.4905 | 0.2670 |
| Smoking Status | -1.6446 | 0.0550 | -0.6527 | 0.5500 |
| Obesity | -1.6660 | 0.0020 | -3.2483 | 0.0000 |
| Underweight | -12.3446 | 0.0920 | -2.4406 | 0.6250 |
| Smoking * lack of recognition | 0.7960 | 0.3380 | 0.8991 | 0.4480 |
| Alcohol * lack of recognition | -1.2010 | 0.1260 | 0.4509 | 0.6710 |
| Smoking * low edu­cational level | 0.0896 | 0.9250 | -0.2591 | 0.8460 |
| Alcohol * low educational level | 0.9864 | 0.2600 | 2.7043 | 0.0240 |
| Mandatory health insurance | -0.0628 | 0.9250 | -0.2444 | 0.7640 |
| Voluntary health insurance | -0.4350 | 0.4970 | -1.6807 | 0.0400 |
| More than 20 minute walk to nearest GP practice (0/1) | -0.1799 | 0.7830 | -0.6934 | 0.3770 |
| More than 20 minute walk to nearest public transport (0/1) | -2.1456 | 0.1900 | 1.6197 | 0.3820 |
| Distance to nearest big city | -0.1714 | 0.2710 | -0.1551 | 0.4370 |
| Crime level | 0.2813 | 0.5130 | -0.5770 | 0.3170 |
| Noise level | -0.5631 | 0.0920 | -0.2540 | 0.5370 |
| Pollution level | -0.0985 | 0.7890 | -0.3441 | 0.4910 |
| Have nobody to confide in (0/1) | -0.0252 | 0.9820 | -1.3311 | 0.3130 |
| Nobody supports career | -0.1464 | 0.7410 | -1.4454 | 0.0150 |
| Autonomy at work | -0.4082 | 0.2050 | -0.1389 | 0.7280 |
| Time pressure at work | 0.3304 | 0.2830 | 0.3042 | 0.4600 |
| No job security | -0.4639 | 0.4640 | 1.2986 | 0.1680 |
| Capability score | 0.4300 | 0.0020 | 0.6215 | 0.0010 |
| Constant | 49.0107 | 0.0000 | 52.2678 | 0.0000 |
| Observation | 1180 |  | 794 |  |
| R-squared | 0.18 |  | 0.16 |  |

Tab. A6
